# Socioeconomic and demographic factors modify observed relationship between caregiving intensity and three dimensions of quality of life in informal adult children caregivers

**DOI:** 10.1186/s12955-018-0996-6

**Published:** 2018-08-29

**Authors:** Sarah K. Cook, Lauren Snellings, Steven A. Cohen

**Affiliations:** 1 Vanderbilt Institute for Clinical and Translational Research, Vanderbilt University Medical Center, 2525 West End Ave, 6th floor, Nashville, TN 3720 USA; 2 Health Studies Program | Department of Kinesiology, University of Rhode Island, 25 W. Independence Way, Kingston, RI 0281 USA

**Keywords:** Caregiver stress, Quality of life, Offspring caregivers, Caregiver burden, Caregiver intensity

## Abstract

**Background:**

The relationship between informal caregiving intensity and caregiver health is well-established, though research suggests this may vary by caregiver demographics. The aim of this exploratory study is to assess the association between caregiving intensity and three dimensions of quality of life outcomes, and determine how caregiver sociodemographics change the nature of this relationship among informal adult children caregivers.

**Methods:**

Using the 2011 National Study of Caregiving, associations between caregiving intensity and quality of life were examined in caregivers providing care to an aging parent (*n* = 1014). Logistic regression was used to model caregiver quality of life on caregiving intensity using an ordinal composite measure of caregiving activities, including Activities of Daily Living (ADL) and Instrumental Activities of Daily Living (IADL), hours per month, and length of caregiving, stratified by race/ethnicity, gender, age, and family income. Odds ratios and corresponding 95% confidence intervals were calculated.

**Results:**

Associations between caregiving intensity and quality of life varied substantially by race/ethnicity, gender, age, and annual family income. White caregivers were significantly more likely to experience negative emotional burden when providing high intensity care (ADL: 1.92, Hours: 3.23). Black caregivers were more likely to experience positive emotions of caregiving (ADL: 2.68, Hours: 2.60) as well as younger caregivers (Hours: 8.49). Older caregivers were more likely to experience social burden when providing high ADL, IADL, and monthly hours of care.

**Conclusions:**

These findings demonstrate the complex and multi-dimensional nature of caregiving, and emphasize the need to develop approaches that are tailored to the specific health needs of subpopulations of informal caregivers.

## Background

In 2014, nearly 34.2 million American adults provided informal care to an adult age 50 years of age or older within the last 12 months, representing approximately 12.4% of the U.S. adult population [1]. Informal caregiving is the unpaid care and support family members and friends voluntarily provide individuals who are unable to function independently. Informal caregivers are estimated to have spent over 30 billion hours a year providing care to disabled or chronically ill individuals, with an opportunity cost of $522 billion per year [2]. These figures are expected to rise as the number of Americans over 65 years of age grows to 72 million by the year 2030 [3].

The need to provide care for this growing segment of the population is a major public health challenge. Numerous studies have shown any type of informal caregiving can result in negative physical and emotional health consequences for the caregiver, often referred to as caregiving-related stress or burden [1, 4–8]. Nearly half of all caregivers who provide care to an older adult over the age of 50 report their caregiving situation is highly stressful [1]. Additionally, caregivers providing high intensity care differ in substantial ways from those providing less care, such as their employment status, the type of caregiving duties they provide, and the impact caregiving has on them [1, 9]. They may be particularly vulnerable to experiencing higher emotional stress, financial strain, and declining health, and quality of life (QOL) [1, 4, 10–16]. With increasing caregiving intensity, the percentages of caregivers reporting fair or poor general health also increases [1]. Higher levels of informal caregiving intensity have specific, negative impacts on aspects of QOL, including emotional health [10, 11, 13] and increase social strain [16].

Additionally, caregiving duties and caregiver health varies by caregiver sociodemographic factors. Differences exist in both the care provided and caregiver QOL among male and female caregivers [11]. Females provide higher levels of informal caregiving than their male counterparts [17], and this disparity has important implications for the health and QOL of female informal caregivers [18–20]. There are also notable disparities in caregiving intensity among caregivers of different racial and ethnic groups [21], ages [10, 22, 23], and income levels [24]. However, it is not well known how associations between caregiving intensity and caregiver QOL vary by these sociodemographic factors. Caregiver race/ethnicity, gender, age, and income may strengthen or weaken this relationship, such that certain caregivers are more or less vulnerable to QOL challenges when providing high intensity care.

Furthermore, “adult child caregivers”, defined as caregivers to a parent or step-parent, are an important subset of caregivers to investigate. They differ from other types of caregivers (i.e. spousal caregivers) in important and distinct ways, such as how they experience the burden of caregiving [7, 25–28]. They may also be particularly prone to strain due to competing responsibilities of employment and providing care for children of their own. As a result of these differences, it has been recommended that adult child caregivers should be examined separately from spousal caregivers, due to significant differences in caregiver characteristics and needs [7, 25].

Therefore it is imperative to better understand how caregiving impacts QOL for different types of adult children caregivers, in order to help guide policies and programs aimed at improving the caregiving experience, ameliorating the associated impact, and improving QOL among this large, important part of the U.S. health care system. To that end, the objective of this exploratory study is to describe and assess the relationship between caregiving intensity and three domains of caregiver QOL (social strain, negative emotional, and positive emotional) in adult children caregivers to older adult parents, and to determine how key sociodemographic variables modify this association. We hypothesize that sociodemographics plays a role in modifying the relationship of caregiving intensity and caregiver QOL.

## Methods

### Study population

The data were obtained from the 2011 National Study of Caregiving (NSOC) dataset, which identified caregivers of National Health and Aging Trends Study (NHATS) participants who were receiving assistance in self-care, mobility, medical, or household activities. NHATS participants were part of a nationally representative cohort of persons age 65 and older who were currently enrolled in Medicare as of 2010. The caregivers of NHATS participants were contacted to participate in a one-time, cross-sectional assessment of caregiving, to include questions on caregiving activities, duration, intensity, and demographics. This analysis focuses on a subset of NSOC participants, adult children caregivers (*n* = 1014). The NSOC data source is unique from prior randomly-sampled, nationally representative surveys because it consists entirely of informal caregivers that were identified by their care recipients.

### Outcome measurements: Caregiver quality of life

Three main outcomes of caregiver QOL were assessed: 1) Social Strain, 2) Negative Emotional Burden, and 3) Positive Emotional Benefit, previously validated through factor analysis [29]. To measure each QOL outcome, items from the NSOC questionnaire pertaining to aspects of caregiver QOL were used (Table 1). Social strain was assessed using participants’ responses to caregiving duties impacting their ability to participate in social activities, such as volunteering, attending religious services, working, and going out for enjoyment. Negative emotional burden was assessed using participants’ responses to questions assessing negative emotional responses, such as anxiousness, worry, depression, and feeling upset. Positive emotional benefit was measured using questions assessing positive emotional responses, such as feeling a sense of purpose, confidence in abilities, cheerfulness, and peace. The top quartile in each domain was coded as ‘High Burden’, while the remaining were coded as ‘Low Burden’.

**Table 1:** Caregiver burden domains and corresponding NSOC questions

Social Strain	
In the last month, did helping [SP] ever … • Keep you from visiting in person with friends or family not living with you? • Keep you from religious services? • Keep you from participating in club meetings or group activities? • Keep you from going out for enjoyment? • Keep you from working for pay? • Keep you from doing volunteer work? • Keep you from providing care for a child or other adult?	
Negative Emotional Burden	
Thinking about the last month, how often did you … . • Feel bored? • Feel lonely? • Feel upset?	
Over the last month, how often have you … . • Had little interest or pleasure in doing things? • Felt down, depressed, or hopeless? • Felt nervous, anxious, or on edge? • Been unable to stop or control worrying?	
• I gave up trying to improve my life a long time ago. • I often feel lonely because I have few close friends.	
• How much does [SP] argue with you? • How often does [SP] get on nervous? • Helping older relatives can be difficult. Is helping [SP] emotionally difficult for you?	
Positive Emotional Benefit	
• My life has meaning and purpose. • In general, I feel confident and good about myself • I like my living situation very much. • I have an easy time adjusting to changes. • I get over (recover from) illness and hardship quickly.	
Thinking about the last month, how often did you … • Feel cheerful? • Feel calm and peaceful? • Feel full of life?	
• How much do you enjoy being with [SP]? • How much does [SP] appreciate what you do for them?	
• Helping [SP] has made you more confident about your abilities. • Helping [SP] has taught you how to deal difficult situations. • Helping [SP] has brought you closer to them. • Helping [SP] gives you satisfaction that they are well cared for.	

*[SP]* NHATS sample person

### Exposure measurements: Caregiver intensity

While there is no unifying measure of caregiving intensity in the caregiving literature, there are well established metrics for measuring caregiving responsibilities and burden. Therefore, four common domains of caregiving were used to assess respondents’ caregiving intensity: 1) Number of Activities of Daily Living (ADLs) performed, 2) Number of Instrumental Activities of Daily Living (IADLs) performed, 3) Hours of caregiving provided a month, and 4) Duration (years) of caregiving. To measure these four intensity domains, items from the NSOC questionnaire assessing aspects of caregiver duties were used. ADLs refer to daily self-care activities that are necessary for fundamental functioning. This was measured by the number of personal care activities caregivers helped with each month, including eating, bathing, dressing, toileting, and helping care recipient move around. IADLs consist of other caregiving activities not necessary for fundamental functioning, but allow an individual to live independently. This domain included the number of instrumental activities caregivers helped their older parent with, including medication management, scheduling medical appointments, and other health and hygiene-related tasks. The last two intensity domains were calculated based on the average number of hours spent caregiving in the last month, and average number of years providing care. The top quartile of each intensity domain was coded as ‘High Caregiving Intensity’, while the rest were coded as ‘Low Caregiving Intensity’. Such an approach was designed to distinguish cases of extreme caregiving from lower levels of caregiving intensity among the population of informal caregivers, which has been identified as an area of concern in previous research [30–32].

The composite measure of caregiving intensity, for each question, those in the “high caregiving intensity” category was counted as a 1, and those in the “low caregiving intensity” were counted as a 0. A sum of these measures for the four questions was calculated. The resultant summed scores ranged from a minimum of 0 (low intensity for all four) to 4 (high intensity for all four measures).

### Demographic modifiers

Demographic characteristics of respondents were assessed using categorical variables, including age, gender, race/ethnicity, and annual family income. Demographic age was categorized into 10-year age groups (< 45, 45–54, 55–64, and 65+). Race/ethnicity was based on three calculated domains (Non-Hispanic White, non-Hispanic Black, and ‘Other’ (Hispanic, American Indian, Native Hawaiian, Pacific Island, other non-Hispanic)). Income was grouped into four $25,000 intervals (≤$24,999, $25,000–$49,999, $50,000–$74,999, and $75,000 or more).

### Statistical analyses

Logistic regression modeling was used to calculate odds ratios (OR) and associated 95% confidence intervals (CI) for the three caregiver QOL outcomes. ORs above 1 indicate increased odds of experiencing caregiver burden among high intensity caregivers, while ORs below 1 indicate decreased odds of experiencing caregiver burden. Overall models were assessed on the entire sample of adult children caregivers, then stratified individually by each of the four potential effect modifier variables. Reference groups were low intensity caregivers within each demographic modifier. Statistical tests for potential effect modification by gender, age, income, and race/ethnicity were also conducted. Models were adjusted for covariates not included as potential moderator variables.

Logistic regression model assumptions were validated in the analysis. First, all dependent variables were binary or ordinal for their appropriate logistic regression model. Second, observations were independent of each other based on data collection methods. For the collinearity assumption, there was minimal but non-negligible correlation among some covariates in some of the stratified models. There was a monotonic association between the outcomes and exposures for most of the stratified models, as well. Model fit was checked using the Bayesian Information Criterion (BIC). For all analyses, the type 1 error rate (alpha) was set to 0.05, and 95% confidence were used, where applicable. SAS version 9.4 (Cary, NC) was used to conduct all statistical analyses.

## Results

### Demographics

The statistics for this sample of adult children caregivers are found in Table 2. The average age of caregivers was 54.6 years old. Sixty-nine percent of respondents were female, while 31% were male. Respondents reported an average annual income of $56,582. Sixty percent of respondents identified as non-Hispanic White, 31% as non-Hispanic Black, and 9% as another racial/ethnic group. Adult children caregivers spent an average of 85 h a month providing care, and had been caring for an elderly parent for 5.6 years. All subsequent results utilized the sample of adult children caregivers and assess the associations between high-intensity caregiving and each of the main sociodemographic exposure variables, stratified by the moderator variables outline below.

**Table 2:** Sample descriptive statistics for NSOC sample of adult-children caregivers

Characteristics	N (%)
Age Group	
< 45	102 (12.6)
45–54	281 (34.7)
55–64	323 (31.9)
≥ 65	104 (12.8)
Average age (years)	54.6
Gender	
Male	310 (30.6)
Female	704 (69.4)
Race/ethnicity	
White, non-Hispanic	610 (60.2)
Black, non-Hispanic	313 (30.9)
Other	91 (9.0)
Annual Income	
< $25,000	206 (29.7)
$25,000-49,999	157 (22.7)
$50,000-74,999	126 (18.2)
≥ $75,000	204 (29.4)
Average annual income	56,582
Average Amount of Care Provided	
Care per month (hours)	85.1
Duration of care (years)	5.6

### Social strain associated with high intensity caregiving

Overall, high ADLs, IADLs, and monthly hours of caregiving were associated with high social strain among caregivers, while duration of caregiving was protective. All results are presented in Table 3.

**Table 3:** Adjusted* odds ratios of caregiver quality of life from high caregiving intensity by race/ethnicity, gender, age, and income

	Social Strain				Negative Emotional Burden				Positive Emotional Benefit			
	Odds Ratio	95% Confidence Interval	*P*-Value	N	Odds Ratio	95% Confidence Interval	*P*-Value	N	Odds Ratio	95% Confidence Interval	*P*-Value	N
**ADLs**												
Overall	**3.89**	**(2.44, 6.21)**	**< 0.001**	**567**	**1.82**	**(1.17, 2.83)**	**0.01**	**567**	0.96	(0.58, 1.56)	0.857	567
**Race/Ethnicity**												
White, NH	**3.85**	**(2.17, 6.81)**	**< 0.001**	**366**	**1.92**	**(1.09, 3.39)**	**0.02**	**366**	0.44	(0.19, 1.02)	0.055	366
Black, NH	**5.04**	**(1.78, 14.31)**	**0.002**	**149**	1.79	(0.76, 4.20)	0.183	149	**2.68**	**(1.20, 6.01)**	**0.02**	**149**
Other	**5.83**	**(1.05, 32.27)**	**0.043**	**52**	2.49	(0.48, 12.98)	0.278	52	0.59	(0.11, 3.27)	0.549	52
**Gender**												
Male	**3.65**	**(1.38, 9.65)**	**0.009**	**196**	1.36	(0.58, 3.17)	0.478	196	0.45	(0.17, 1.22)	0.118	196
Female	**3.76**	**(2.20, 6.44)**	**< 0.001**	**371**	**1.96**	**(1.16, 3.31)**	**0.01**	**371**	1.30	(0.72, 2.33)	0.384	371
**Age**												
< 45 years	1.79	(0.37, 8.75)	0.472	79	0.75	(0.23, 2.50)	0.642	71	1.63	(0.47, 5.66)	0.439	79
45–54 years	**3.30**	**(1.48, 7.33)**	**0.003**	**200**	**3.14**	**(1.47, 6.72)**	**0**	**200**	1.64	(0.77, 3.50)	0.204	200
55–64 years	**5.56**	**(2.65, 11.65)**	**< 0.001**	**223**	1.61	(0.78, 3.30)	0.196	223	0.42	(0.14, 1.28)	0.125	223
65+ years	4.07	(1.00, 16.58)	0.051	65	2.75	(0.48, 15.84)	0.258	65	0.41	(0.08, 2.14)	0.289	65
**Income**												
$0–24,999	**2.68**	**(1.16, 6.18)**	**0.021**	**174**	1.53	(0.78, 3.02)	0.218	174	1.10	(0.51, 2.36)	0.810	174
$25,000-49,999	**3.40**	**(1.37, 8.46)**	**0.008**	**130**	1.52	(0.58, 3.97)	0.398	130	1.20	(0.43, 3.40)	0.726	130
$50,000-74,999	2.85	(0.81, 10.05)	0.103	94	1.46	(0.43, 5.01)	0.547	94	0.48	(0.10, 2.35)	0.362	94
$75,000 +	**6.46**	**(2.50, 16.73)**	**< 0.001**	**169**	2.71	(0.98, 7.95)	0.054	169	0.88	(0.30, 2.62)	0.822	169
**IADLs**												
Overall	**2.90**	**(1.80, 4.67)**	**< 0.001**	**567**	1.48	(0.94, 2.35)	0.091	567	1.36	(0.85, 2.20)	0.202	567
**Race/Ethnicity**												
White, NH	**2.40**	**(1.32, 4.37)**	**0.004**	**296**	1.60	(0.88, 2.92)	0.123	366	1.16	(0.57, 2.37)	0.675	366
Black, NH	2.27	(0.86, 6.00)	0.099	128	1.42	(0.59, 3.38)	0.432	149	1.73	(0.78, 3.86)	0.178	149
Other	**31.54**	**(3.30, 301.6)**	**0.003**	**44**	1.50	(0.35, 6.47)	0.591	52	1.53	(0.40, 5.80)	0.531	52
**Gender**												
Male	2.12	(0.75, 6.00)	0.157	174	1.04	(0.40, 2.67)	0.940	196	1.53	(0.65, 3.62)	0.335	196
Female	**3.15**	**(1.83, 5.42)**	**< 0.001**	**294**	1.64	(0.97, 2.79)	0.065	371	1.27	(0.71, 2.27)	0.416	371
**Age**												
< 45 years	1.55	(0.32, 7.49)	0.584	79	1.59	(0.50, 5.02)	0.429	79	6.22	(1.74, 22.15)	0.01	79
45–54 years	2.23	(0.98, 5.06)	0.055	200	1.87	(0.84, 4.20)	0.127	200	1.39	(0.62, 3.10)	0.429	200
55–64 years	**3.56**	**(1.68, 7.52)**	**0.001**	**223**	1.04	(0.49, 2.21)	0.913	223	0.75	(0.29, 1.89)	0.536	223
65+ years	**5.34**	**(1.08, 26.28)**	**0.040**	**65**	4.40	(0.72, 26.84)	0.109	65	1.23	(0.25, 6.05)	0.803	65
**Income**												
$0–24,999	**3.09**	**(1.35, 7.09)**	**0.008**	**174**	1.64	(0.83, 3.23)	0.157	174	1.15	(0.53, 2.47)	0.723	174
$25,000-49,999	2.50	(0.94, 6.67)	0.068	130	1.23	(0.41, 3.87)	0.718	130	1.90	(0.60, 5.97)	0.274	130
$50,000-74,999	2.46	(0.65, 9.29)	0.184	94	**4.02**	**(1.11, 14.52)**	**0.03**	**94**	1.81	(0.52, 6.35)	0.352	94
$75,000 +	**4.25**	**(1.56, 11.54)**	**0.005**	**169**	0.72	(0.19, 2.72)	0.630	169	1.45	(0.56, 3.78)	0.450	169
**Hours of caregiving per week**												
Overall	**4.88**	**(2.91, 8.19)**	**< 0.001**	**562**	**2.57**	**(1.60, 4.13)**	**< 0.001**	**562**	1.42	(0.85, 2.36)	0.182	562
**Race/Ethnicity**												
White, NH	**7.49**	**(3.82, 14.68)**	**< 0.001**	**362**	**3.23**	**(1.69, 6.18)**	**< 0.001**	**362**	0.73	(0.29, 1.83)	0.497	362
Black, NH	**2.93**	**(1.11, 7.76)**	**0.031**	**148**	1.98	(0.85, 4.57)	0.111	148	2.60	(1.19, 5.69)	0.02	148
Other	1.96	(0.32, 12.01)	0.469	52	1.98	(0.44, 8.96)	0.375	52	1.26	(0.27, 5.77)	0.769	52
**Gender**												
Male	**4.93**	**(1.66, 14.58)**	**0.004**	**194**	**2.98**	**(1.20, 7.38)**	**0.02**	**194**	0.45	(0.15, 1.37)	0.160	194
Female	**4.69**	**(2.59, 8.50)**	**< 0.001**	**368**	**2.36**	**(1.35, 4.12)**	**0**	**368**	**2.17**	**(1.19, 3.93)**	**0.01**	**368**
**Age**												
< 45 years	1.97	(0.39, 9.91)	0.412	79	0.76	(0.22, 2.66)	0.668	79	**8.49**	**(2.19, 32.96)**	**0**	**79**
45–54 years	**4.07**	**(1.68, 9.84)**	**0.002**	**199**	**4.73**	**(2.06, 10.88)**	**< 0.001**	**199**	1.84	(0.80, 4.21)	0.149	199
55–64 years	**7.77**	**(3.38, 17.87)**	**< 0.001**	**220**	2.13	(1.00, 4.57)	0.051	220	**0.27**	**(0.07, 0.99)**	**0.05**	**220**
65+ years	5.05	(1.00, 25.60)	0.050	64	**8.43**	**(1.25, 57.06)**	**0.029**	**64**	1.37	(0.27, 7.03)	0.706	64
**Income**												
$0–24,999	**3.45**	**(1.45, 8.21)**	**0.005**	**173**	**2.75**	**(1.40, 5.41)**	**0**	**173**	1.96	(0.95, 4.08)	0.071	173
$25,000-49,999	**11.46**	**(3.75, 34.97)**	**< 0.001**	**129**	2.08	(0.69, 6.27)	0.195	129	0.71	(0.18, 2.78)	0.628	129
$50,000-74,999	**9.99**	**(2.18, 45.76)**	**0.003**	**94**	4.04	(0.93, 17.61)	0.063	94	0.84	(0.14, 4.88)	0.842	94
$75,000 +	3.16	(0.99, 10.07)	0.052	166	1.60	(0.40, 6.37)	0.508	166	1.25	(0.37, 4.18)	0.721	166
**Years of caregiving**												
Overall	**0.56**	**(0.33, 0.97)**	**0.040**	**498**	1.23	(0.77, 1.96)	0.380	498	0.78	(0.48, 1.26)	0.308	498
**Race/Ethnicity**												
White, NH	0.64	(0.33, 1.28)	0.206	325	1.39	(0.76, 2.56)	0.285	325	0.67	(0.33, 1.36)	0.266	325
Black, NH	0.48	(0.17, 1.38)	0.172	129	1.10	(0.46, 2.61)	0.836	129	0.68	(0.30, 1.54)	0.349	129
Other	0.29	(0.03, 2.96)	0.294	44	1.00	(0.22, 4.54)	0.996	44	1.48	(0.35, 6.27)	0.597	44
**Gender**												
Male	0.32	(0.10, 1.07)	0.064	181	1.07	(0.45, 2.54)	0.887	181	0.94	(0.43, 2.06)	0.885	181
Female	0.65	(0.35, 1.22)	0.179	317	1.31	(0.75, 2.27)	0.342	317	0.65	(0.34, 1.22)	0.181	317
**Age**												
< 45 years	–	–	0.998	67	0.80	(0.24, 2.68)	0.713	67	1.58	(0.42, 5.88)	0.497	67
45–54 years	1.01	(0.43, 2.39)	0.985	174	1.59	(0.70, 3.60)	0.268	174	0.69	(0.30, 1.59)	0.384	174
55–64 years	**0.32**	**(0.12, 0.83)**	**0.019**	**203**	0.99	(0.49, 2.00)	0.987	203	0.56	(0.24, 1.33)	0.190	203
65+ years	1.73	(0.39, 7.77)	0.475	54	2.52	(0.29, 21.98)	0.403	54	1.31	(0.33, 5.26)	0.706	54
**Income**												
$0–24,999	**0.36**	**(0.14, 0.93)**	**0.035**	**146**	1.13	(0.56, 2.29)	0.727	146	0.62	(0.27, 1.42)	0.256	146
$25,000-49,999	0.28	(0.07, 1.04)	0.058	112	1.35	(0.45, 4.06)	0.590	112	1.40	(0.50, 3.93)	0.527	112
$50,000-74,999	1.18	(0.26, 5.39)	0.828	85	1.39	(0.32, 6.12)	0.662	85	0.74	(0.21, 2.65)	0.644	85
$75,000 +	0.98	(0.37, 2.63)	0.972	155	1.47	(0.53, 4.11)	0.458	155	0.62	(0.24, 1.61)	0.324	155

Bolded results are statistically significant (*p* < 0.05)

*Adjusted for marital status, co-residence with care recipient, and each of the effect modifiers except the one being used in the individual effect modification model. For example, for the models stratified by income category - age, gender, and race/ethnicity are treated as confounders

#### Race/ethnicity

The association between caregiving intensity and social strain varied significantly by caregiver race/ethnicity. Black, non-Hispanic and ‘Other’ caregivers providing high ADL care had greater odds of experiencing social burden than their counterparts providing low ADL care (OR = 5.04, CI [1.78, 14.31] and OR = 5.83, CI [1.05, 32.27] respectively). White non-Hispanic caregivers providing high ADL care were also at higher odds of experiencing social strain (OR = 3.85, CI [2.17, 6.81]).. A significant association was also observed for high IADL caregiving among ‘Other’ caregivers (OR = 31.54, CI [3.30, 301.6]) and White, non-Hispanic caregivers (OR = 2.40, CI [1.32, 4.37]). High number of monthly hours of caregiving was predictive of social burden among White, non-Hispanics (OR = 7.49, CI [3.82, 14.68]) and Black, non-Hispanic caregivers (OR = 2.93, CI [1.11, 7.76]).

#### Gender

Females providing high IADL care were 3.15 times as likely to report experiencing social strain compared to low IADL females (CI [1.83, 5.42]).

#### Age

Generally, the oldest high intensity caregivers (> 45 years) were at greater odds of experiencing social strain. Caregivers between the age of 45–54 and 55–64 years providing high ADL care were 3.30 times (CI [1.48, 7.33]) and 5.56 times (CI [2.65, 11.65]), respectively, as likely to report experiencing high social strain compared to their low caregiving counterparts. Similar results were observed for caregivers providing high monthly hours of care: 45–54 years (OR = 4.07, CI [1.68, 9.84]) and 55–64 years (OR = 7.77, CI [3.38, 17.87]). For high IADL caregiving, the oldest caregivers were more likely to report experiencing social strain compared to their low IADL caregiving counterparts: 55–64 years (OR = 3.56 CI [1.68, 7.52]), 65+ years (OR = 5.34, CI [1.08, 26.28]). However, high number of years providing care was protective of experiencing social strain in caregivers 55–64 years of age (OR = 0.32, CI [0.12, 0.83]).

#### Annual income

High ADL caregiving was associated with social strain in caregivers earning at least $75,000 a year (OR = 6.46, CI [2.50, 16.73]). Caregivers in the lowest (less than $25,000) and highest ($75,000+) income bracket providing high IADL care had greater odds of reporting social strain than low intensity caregivers: OR (3.09), CI [1.35, 7.09] and OR (4.25), CI [1.56, 11.54] respectively. Caregivers providing a high number of monthly hours of care had significantly higher odds of reporting social strain in both the $25,000–49,999 (OR = 11.46, CI [3.75, 34.97]) and $50,000–74,999 (OR = 9.99, CI [2.18, 45.76]) subgroups. Caregivers in the lowest income bracket providing care for a high number of years were less likely to experience social strain (OR = 0.36, CI [0.14, 0.93]) than their counterparts providing care for a lesser number of years.

### Emotional QOL: Negative and positive emotions associated with high intensity caregiving

#### Race/ethnicity

White, non-Hispanic caregivers providing high ADL and high number of monthly hours of care had higher odds of reporting negative emotional burden than their low intensity counterparts: ADL (OR = 1.92, CI [1.09, 3.39]) and monthly hours of care (OR = 3.23, CI [1.69, 6.18]). Black non-Hispanic high intensity caregivers were more likely to report experiencing positive emotions associated with caregiving: ADL (OR = 2.68, CI [1.20, 6.01]) and monthly hours of care (OR = 2.60, CI [1.19, 5.69]).

#### Gender

Females providing high ADL care had greater odds of experiencing negative emotions (OR = 1.96, CI [1.16, 3.31]) than females providing low ADL care. When providing a high number of monthly hours of caregiving, males were more likely to report experiencing negative emotional burden (OR = 2.98, CI [1.20, 7.38]), while females were more likely to experience positive emotional benefit from caregiving (OR = 2.17, CI [1.19, 3.93]).

#### Age

Caregivers between the age of 45 and 55 years old providing high ADL care were 3.14 times as likely to experience negative emotions associated with caregiving (CI [1.47, 6.72]) than their low intensity caregiver counterparts. When providing a high number of monthly hours of care, this same group was also more likely to report negative emotional burden (OR = 4.73, CI [2.06, 10.88]), as were the oldest caregivers (65+ years): OR (8.43), CI [1.25, 57.06]). The youngest caregivers (< 45 years of age) were the only group to report experiencing positive emotional benefits of caregiving. Those providing high IADL care and a high number of monthly hours of care were significantly more likely to report experiencing positive emotions than low intensity caregivers: IADL (OR = 6.22, CI [1.74, 22.15]) and monthly hours (OR = 8.49, CI [2.19, 32.96]). Caregivers between the ages of 55 and 64 years providing high monthly hours of care had lower odds of experiencing positive caregiving emotions (OR = 0.27, CI [0.07–0.99])) compared to those providing low monthly hours of care.

#### Annual income

Caregivers earning between $50,000 and $74,999 a year were more likely to experience negative emotional burden when providing high IADL care (OR = 4.02, CI [1.11, 14.52]) and high monthly hours of care (OR = 4.04, CI [0.93, 17.61]) compared to those providing low intensity care.

## Discussion

The findings of this study suggest that caregiver demographics substantially change the nature of the association between caregiving intensity and caregiver quality of life. These results provide insight into how informal adult-children caregivers are affected by the caregiving experience, and suggestive evidence for intentionally tailoring policies and programs to the needs and values of specific caregiver groups involved in different manifestations of high intensity caregiving. Our findings indicate that the experience of providing informal care to an adult parent is often complex and multidimensional, affecting people in distinct ways. This ‘unique caregiving fingerprint’ demonstrates how socioeconomic and demographic characteristics of the caregiver has critical implications for how their caregiving responsibilities impact them.

Our results indicate that older caregivers (45 years of age or older) are more likely to experience negative consequences of caregiving – especially social burden – than their younger caregiving counterparts (less than 45 years of age). Younger caregivers are also more likely to experience the positive emotional aspects of caregiving, when providing both high ADL and high number of monthly hours of care. Additionally, the results suggest that younger caregivers may be more emotionally and psychologically resilient than older caregivers, as younger caregivers were less vulnerable to negative emotional burden while simultaneously more likely to experience the positive emotions associated with providing care. This finding is counter to previous studies that have noted worse physical and mental health in younger caregivers.

One study examining data from the 2000 Behavioral Risk Factor Surveillance System noted that younger caregivers were at an increased risk for experiencing fair or poor health compared to their non-caregiving counterparts, while older caregivers had a decreased risk [22]. These younger caregivers also showed larger deficits in both mental and physical health and QOL measures compared to older caregivers. A second study by Anderson et al. [23] found that younger caregivers were significantly more likely to report experiencing mental distress and being dissatisfied with life compared to older caregivers, but were less likely to report fair or poor health or physical distress. Other studies have found younger age independently predicts depression among caregivers [10]. Classification of younger and older caregiver groups varied, making it difficult to compare and interpret results across studies. Nevertheless, our findings are in contrast to prior research and suggest that the youngest informal caregivers may be especially psychologically resilient and less likely to experience emotional caregiver burden.

Differences in emotional well-being that varied across racial/ethnic groups were also observed. Consistent with previous studies, White caregivers were more likely to report negative emotional burden from high intensity caregiving than caregivers of other race/ethnicities. African American caregivers were not at an increased risk for negative emotional burden, but were more likely to experience the positive emotional aspects of caregiving. This adds to the growing body of literature noting racial differences in emotional adaptation of informal caregivers, with White caregivers being particularly vulnerable to reporting emotional distress, and African American caregivers demonstrating emotional resilience, despite both groups providing highly involved care. Several studies have observed lower levels of psychological distress among African American caregivers, highlighting their increased ability to emotionally adapt to the stresses of providing care for family members [33–35]. It is speculated that the positive perceptions associated with caregiving, combined with one’s own resourcefulness may buffer against the impact of negative emotional distress, such as anxiety, depression, and hostility [36]. A meta-analysis examining the ethnic differences in psychological outcomes of family caregivers found that some ethnic minority caregivers were at an advantage with regard to psychological health, but disadvantaged in physical health outcomes [17]. This was primarily observed in African Americans, with inconsistent findings among Hispanics, and worse psychological outcomes in Asian-American caregivers. While our study did not assess physical health outcomes, Pinquart and Sörensen note their findings may be due to caregiving’s relatively small impact on overall physical health, and more of a reflection of the social conditions and determinants of health that often vary along racial lines, including health insurance coverage, health care access, and racial discrimination [17].

While our findings reveal patterns in caregiver burden across certain age and racial/ethnic groups, it is difficult to discern any overarching themes across other demographics. Parsing out patterns in caregiving burden are especially difficult when stratified by caregiver income, as there appear to be few that are distinguishable, and even conflicting results. For instance, caregivers providing high ADL caregiving were more likely to experience social strain than their low-intensity caregiving counterparts in all income brackets except $50,000-74,999. Previous research has noted higher degrees of burnout among lower income caregivers [37, 38], and we would anticipate this same pattern across all income categories in this domain.

Our results also demonstrate to some degree, the high prevalence and commonly shared experience of social strain among all caregivers – the act of providing care and caregiving responsibilities impacting one’s ability to engage and participate in social activities. This finding is consistent with other studies. Haley et al. note that both African American and Caucasian caregivers experience similar social consequences, with restrictions of social activities as a result of caregiving duties [37]. While our findings do indicate that certain groups of caregivers may be at an even heightened risk, the vast majority of all high intensity caregivers reported experiencing social strain as a result of their care responsibilities. This finding alone may indicate the need to find solutions in helping to alleviate high intensity caregiving’s impact on caregivers’ ability to engage socially within society. Interestingly, duration (years) of caregiving was the only caregiving intensity measure that was protective of experiencing social strain, significant in both 55–64 year old caregivers and caregivers in the lowest income bracket (less than $25,000/year).

Lastly, our findings demonstrate that the measurement of caregiving intensity matters. Different measures of intensity and ways people provide care (ADL, IADL, Hours, and Years providing care) impact the observed associations between caregiving intensity and QOL outcomes. If caregiving intensity is measured by the number of hours spent providing care, we find a substantial amount of negative emotional burden among caregivers. However, if intensity is measured by IADLs performed, only a weak association between caregiving intensity and negative emotional aspects of caregiving is observed. Likewise, duration of caregiving demonstrated to be a relatively poor predictor of caregiver emotional burden. Therefore, associations between intensity and caregiver quality of life vary substantially based on the measurement of caregiving intensity used, and should be taken into account in future studies. Such findings are consistent with previous research suggesting that the choice of caregiving intensity measure changes its association with health and QOL outcomes. Few studies have directly compared measures of caregiving intensity and the potential for differences in aspects of caregiving intensity to impact health. One study found that household income was associated with hours per month spent caregiving, but not associated with number of ADLs or IADLs with which the caregiver provided care [39]. A study of caregiving intensity and fall risk used individual ADL or IADL tasks and found that the performing housework or other home maintenance work was protective against falls, while no other ADL or IADL was significantly associated with fall risk. The same study found that daily or more frequent caregiving was associated with an increased fall risk [40]. Other studies have assessed caregiving intensity using number of ADLs caregivers performed for their care recipient [41, 42].

### Strengths and limitations

When interpreting these findings, there are several important limitations to note. First, due to the cross-sectional nature of this study, we are unable to determine causal relationships between caregiving intensity and caregiver burden. These associations have been well documented in the literature, however less is known about the causal nature of caregiving. It is possible that caregiver duties are responsible for caregiver stress, or perhaps having an ill, elderly parent contributes to caregiver burden, irrespective of caregiving duties. Second, all exposure and outcome measures were dichotomized as high/low. Important distinctions and variabilities in the caregiver experience may have been lost due to collapsing these variables and selection of the cut-points for the categorization. Future research should consider using a continuous caregiver index, or perhaps include moderate levels of caregiving intensity and burden to address this limitation and decipher if important differences exist among caregiving intensities. Third, the small sample size of some of the demographic groups likely limited the ability to observe significant associations. Next, although the caregiver QOL outcomes have been validated in multiple demographic subgroups previously [29], the use of these three specific domains does not fully address the wide scope of QOL issues that arise from informal caregiving duties. Future studies can consider other aspects of caregiver QOL in addition to the social and emotional domains assessed in this study. Similarly, the caregiver intensity variables (ADLs, IADLs, hours per month) have not yet been fully validated in the literature, as there lacks consensus as to the best measures of caregiving intensity.

Another important limitation is the treatment of gender as a sociodemographic characteristic in the analysis. Gender itself is multidimensional and has important implications in terms of gender roles, norms, and stereotypes. Furthermore, gender and other social identities such as race/ethnicity, place of residence, employment status, and others may intersect or combine to affect the QOL of informal caregivers [43, 44]. Future research should explore these gender roles, norms, and stereotypes and their intersections with other social identities such as race/ethnicity and employment simultaneously in the context of impacting caregiver health and QOL. A final limitation is the focus of this analysis on adult children caregivers, a specific sub-set of caregivers. Therefore, the findings may not be generalizable to a broader caregiver population. The experience and needs of adult children caregivers may be quite different from those of other informal adult caregivers, such as those providing care for a spouse, child, or other family member or friend. However adult children caregivers still represent a very important and large segment of the caregiving community. According to a recent report, 47% of caregivers providing care for an adult over the age of 50 years were caring for a parent [1]. This group is only expected to grow as the US sees a dramatic shift in the aging and demographic structure of older adults.

Despite these limitations, this study has a number of strengths. First, this study uses a nationally representative sample of caregivers identified by their care recipients. Second, all caregiving QOL domains were previously identified by factor analysis, due to lack of a unified measure. These three separate burden domains allowed for a comprehensive assessment of caregiver intensity and QOL outcomes. Lastly, to the best of our knowledge, this is the first time this particular dataset has been analyzed in such a way as to better decipher the relationship between caregiving intensity and QOL outcomes.

## Conclusion

Our findings from this study indicate that demographic characteristics of informal adult-children caregivers have an important impact on the caregiver experience. By changing the nature of how caregivers are affected by their caregiver responsibilities (here, defined as ‘caregiving intensity’), sociodemographic information helps us to better understand how different groups of caregivers may be disproportionately impacted and vulnerable to experiencing various types of caregiving stress.

While this exploratory study’s findings draw important associations, more research is needed to delve deeper into better understanding the needs of different caregivers, as well as elucidate the relationships and often inconsistent findings that were observed. Qualitative methods of inquiry may be useful in explaining the ‘hows’ and ‘whys’ behind these findings, as well as help to gain a better understanding of the specific needs, values, and ways to effectively intervene in order to improve caregiver health. These findings may be valuable for those in a position to develop programs, policies, and interventions that are appropriately aligned with the specific needs of individual groups of informal caregivers. These strategies should be informed by research and rigorously tested and evaluated to insure they are effective at meeting the needs of the caregivers they are intended to serve.

## Abbreviations

ADLs: Activities of Daily Living
IADLs: Instrumental Activities of Daily Living
NHATS: National Health and Aging Trends Study
NSOC: National Study of Caregiving
QOL: Quality of Life
SP NHATS: sample person
